# Spatial Transcriptomics, Proteomics, and Epigenomics as Tools in Tissue Engineering and Regenerative Medicine

**DOI:** 10.3390/bioengineering11121235

**Published:** 2024-12-06

**Authors:** Mikko J. Lammi, Chengjuan Qu

**Affiliations:** 1 Institute of Medical and Translational Biology, Umeå University, 90187 Umeå, Sweden; 2 Department of Odontology, Umeå University, 90187 Umeå, Sweden; chengjuan.qu@umu.se

**Keywords:** single-cell RNA sequencing, spatial multiomics, bioengineering, regenerative medicine

## Abstract

Spatial transcriptomics, proteomics, and epigenomics are innovative technologies which offer an unparalleled resolution and wealth of data in understanding and the interpretation of cellular functions and interactions. These techniques allow researchers to investigate gene and protein expressions at an individual cell level, revealing cellular heterogeneity within, for instance, bioengineered tissues and classifying novel and rare cell populations that could be essential for the function of the tissues and in disease processes. It is possible to analyze thousands of cells simultaneously, which gives thorough insights into the transcriptomic view of complex tissues. Spatial transcriptomics combines gene expressions with spatial information, conserving tissue architecture and making the mapping of gene activity across different tissue regions possible. Despite recent advancements in these technologies, they face certain limitations. Single-cell transcriptomics can suffer from technical noise and dropout events, leading to incomplete data. Its applicability has been limited by the complexity of data integration and interpretation, although better resolution and tools for the interpretation of data are developing fast. Spatial proteomics and spatial epigenomics provide data on the distribution of proteins and the gene regulatory aspects in tissues, respectively. The disadvantages of these approaches include rather costly and time-consuming analyses. Nevertheless, combining these techniques promises a more comprehensive understanding of cell function and tissue organization, which can be predicted to be useful in achieving better knowledge of cell guidance in tissue-engineered constructs and a higher quality of tissue technology products.

## 1. Introduction

Tissue engineering and translational regenerative medicine are fields that aim to restore, maintain, or enhance tissue and/or organ function. They apply the principles from cell biology, medicine, genetics, engineering, and material science in order to develop biological substitutes to replace damaged or malfunctioning tissues and organs [1]. Regenerative medicine further aims to support the body’s natural healing processes to regenerate and repair tissues. This approach often combines stem cells, biomaterials, and soluble factors, such as growth factors, and increasingly incorporates three-dimensional bioprinting [2].

New treatments are being developed in these areas, from repairing damaged heart muscle after a heart attack to growing new nerves in spinal cord injuries and regenerating injured brain tissue, to name a few. Advances in biomaterials, stem cell technologies, and three-dimensional bioprinting enable the generation of tissue structures that closely mimic the native structures. The ultimate goals of tissue engineering are to repair or replace damaged tissues and organs, restore lost functions, and produce functional, biologically compatible, and transplantable tissues and organs grown in the laboratory that can integrate with the body. This would reduce the reliance on human donor organs and improve patient outcomes. For this, challenges in ensuring biocompatibility, vascularization, innervation, the handling of inflammation, and the functional integration of engineered tissues remain to be solved to fully reach the potential of these technologies [3].

The primary purpose of using omics techniques in tissue engineering and regenerative medicine is to verify the functionality, precision, reproducibility, and reliability of tissue- and organ-specific features for clinical diagnostics and therapies. Detailed molecular profiling can identify the unique molecular signatures of specific tissues and organs. In engineered tissues, spatial omics can be used to confirm that the arrangement of cell types and their molecular profiles mirror those of native tissues, ensuring suitability for clinical use. Additionally, spatial omics can validate whether bioengineered tissues accurately model disease patterns, which is crucial for drug discovery and testing.

Omics techniques could play a crucial role, for instance, in validating bioengineered liver tissue for clinical applications. They could be used for ensuring functionality by analyzing liver-specific gene expressions through transcriptomics and verifying proper protein production. Additionally, these techniques can assess safety by monitoring inflammation or fibrosis, and evaluate reproducibility, such as proper liver zonation and integration. They also support tracking functionality and safety in preclinical models.

Spatial omics can help to ensure clinical applicability by mapping molecular features in tissues to assess functionality, structure, and integration. For bioengineered hearts, this includes confirming cardiac gene and protein expression, verifying regional organization, detecting inflammation or fibrosis markers, ensuring consistency across batches, and tracking host–tissue interactions.

Quality control is a critical component of tissue-engineered transplantable tissue to ensure strict safety, efficacy, and performance standards before clinical use. The quality control processes include many steps, such as cell sourcing and characterization and purity, biomaterial selection and testing, manufacture process control, functional evaluation, in vivo biological performance testing, sterility and safety testing, the host immune response, regulatory compliance, and batch-to-batch consistency.

Since the first single-cell transcriptomic analysis in 2009 [4], technologies for studying cells at the single-cell level have advanced rapidly. These tools allow researchers to characterize cell populations, their behavior in tissues, and the functional properties of specific cell types [5]. Possible temporal differentiation and developmental changes in the cells in potential transplants can be observed by single-cell transcriptomics, and spatial methods can verify the desired distribution and character of the cells in the manufactured tissues.

## 2. Single-Cell Transcriptomics

DNA is transcribed into messenger RNAs, which are further translated into proteins. Some DNA also produces non-coding RNAs, such as transfer RNAs, ribosomal RNAs, and microRNAs, that help in protein synthesis and gene regulation. Today, the most commonly used methods analyzing total RNA transcripts provide an overall gene expression profile or average expression, while single-cell RNA analysis gives detailed, high-resolution insights into gene expression within individual cells and can even detect different, possibly new, cell types in the samples.

The principle of single-cell RNA sequencing involves the isolation and analysis of the complete set of RNA transcripts from individual cells, uncovering the distinct profiles of different cell types within a complex tissue. Various technologies are available for single-cell transcriptomics which have their own strengths and weaknesses. Droplet-based microfluidics uses encapsulated cells in droplets with unique barcodes to enable the identification of individual cells. Its advantages are its high throughput, relatively low cost, and minimal cell loss. In microwell-based platforms, cells are captured in microwells, each with unique barcodes. This technology provides high-resolution spatial information and flexibility in cell types. In plate-based systems, cells are lysed and processed in the individual wells of a plate. This technology has a relatively simple workflow.

In droplet-based microfluidics, the analysis begins with the isolation of the cells from a tissue sample or cell population using techniques such as fluorescence-activated cell sorting, microfluidics, or laser-capture microdissection. Gel beads barcoded with a unique DNA oligonucleotide can be used to capture single cells. Microfluidics is used to form emulsions of droplets, each of which ideally contains one cell and one barcoded gel bead. In each drop, the cell is lysed to release its RNA content, and the gel bead dissolves to release the barcoded primers. This step takes place inside the droplets, ensuring that each cell’s RNA is captured with a unique barcode specific to that gel bead. The released RNA molecules hybridize with the barcoded primers, and reverse transcription to cDNA is performed in the droplets, producing cDNA labeled with unique cell-specific barcodes and unique molecular identifiers. After cDNA synthesis, the amplified cDNA is prepared into a sequencing library and sequenced using next-generation sequencing. Bioinformatics tools are used to process raw data, align the reads to a reference genome, quantify gene expression levels, and identify unique transcript sequences. After that, various advanced computational methods are used to interpret the data.

## 3. Spatial Transcriptomics

Spatial transcriptomics technology enables the mapping of the spatial distribution of gene expression in tissues and reveals the relationship between gene function and tissue architecture [6]. Today, this technique allows multicellular, unicellular, and even subcellular resolution between nuclear and cytoplasmic transcripts, mainly depending on the technique used and the resolution of the device [7]. A technique named Seq-cope can achieve a submicrometer resolution (≈0.6 μm on average) and good pixel density, making nuclear–cytoplasmic transcriptome architecture from tissue sections possible [8]. Sample sources can be, for instance, animal/human tissues, biofilms, cell cultures, or organoids, as well as both frozen and formalin-fixed paraffin sections. The methods by which mRNA molecules obtain their spatial barcodes differ somewhat for frozen and formalin-fixed samples. Importantly, spatially distinguishable tissue cell types, cell states, histories, and predicted fates can be obtained from the large amount of data collected from the samples. Simultaneous progress in the computing power of computers and the development of software tools to analyze and present huge amounts of raw data have paved the way for better and easier interpretation of the data [9].

A new advancement in spatial transcriptomics was achieved with the creation of a genome-wide three-dimensional atlas from the single-cell sequencing data of cryosections [10]. Consecutive 10 μm sections covering a 350 μm tissue depth were able to capture the spatial cell-type complexity in humans’ healthy and metastatic lymph nodes [11].

## 4. Spatial Proteomics

Spatial proteomics aims to map the location and distribution of proteins in organs, tissues, and even cells by combining proteomics techniques with imaging methods [12]. It can provide researchers with several advances in cell and tissue biology. These can be related to the understanding of protein localization (subcellular, cellular, and spatial organization), protein interactions, and cellular processes and networks, and they can help to find spatial biomarkers. Today, it makes, for instance, the construction of organ tissue spatial atlases possible [13].

Spatial proteomics can involve the use of antibody-related imaging methods analyzing protein distribution and interactions with spatial resolution, especially utilizing fluorescence microscopy, so that even the imaging of single-molecule localization can be possible. Multiplexed imaging allows the analysis of dozens to hundreds of proteins. In diseases, the mislocation or new expression of a protein(s) can indicate the onset of various diseases, which could be detected by spatial proteomics.

Recent advances in mass-spectrometry-based proteomics have overcome significant technical challenges, enabling highly sensitive and reproducible measurements of protein distribution at high spatial resolutions. Manual tissue microdissection combined with mass-spectrometry-based proteomic analysis could assess tissue’s spatial variation in a proteome at a 125–160 μm lateral resolution [14].

## 5. Spatial Epigenetics

Epigenetic modifications and the physical proximity of nucleic acids are decisive factors in the control of biological functions and the development of diseases. They regulate the degree of DNA packaging and how accessible it is to transcriptional machinery, thereby controlling whether genes are activated or silent. DNA methylation and histone modifications play key roles in this regulatory process. Current cell imaging techniques, such as fluorescence, in situ hybridization, and immunofluorescence, have suffered from insensitivity in detecting low-level DNA modifications and their spatial relationships. However, with advanced techniques, it is possible to obtain mappings of histone modifications and the profiling of chromatin states in tissue sections with a good spatial resolution [15]. A transposase enzyme is used to insert sequencing adapters into accessible, open DNA regions, and DNA is then sequenced to allow the mapping of the open chromatin. Spatially mapped single-cell chromatin accessibility data can provide multidimensional insights into the regulatory landscape of tissues and their cellular organization [16].

## 6. Conclusions

Taken together, spatial transcriptomic, proteomic, and epigenomic techniques provide valuable tools for understanding the spatial organization and function of tissues. These techniques produce large datasets that require special computational tools to analyze and interpret the data. Despite the advances in these techniques, there are still challenges to their widespread application. Currently, the high costs and strict sample requirements of single-cell technologies may scare off many researchers [17]. As with all developing technologies, these technologies can be expected to develop in such a way that their costs and availability improve with the expansion of the user base of researchers interested in them.

Spatial transcriptomics can capture different gene expression profiles of different cell types in a tissue and provide information on the spatial distribution of gene expression which is important for understanding tissue organization and function. This enables the identification of cell-type-specific markers and pathways. Still, their sensitivity may be limited in detecting low-abundance transcripts, and the spatial resolution can be still improved.

In addition to providing information on the spatial distribution and abundance of proteins, spatial proteomics can also identify post-translational modifications that can significantly change protein functions. It still has limitations in sensitivity for low-abundance proteins and may require complex sample preparation techniques.

Spatial epigenetics reveals the epigenetic mechanisms that regulate gene expression and provides insight into the epigenetic basis of diseases. Thus, it may identify potential therapeutic targets for epigenetic diseases. However, due to the complexity of epigenetic changes, the results are often difficult to interpret. The techniques may also have limitations in spatial resolution.

Thus, bioengineering provides platforms which can be analyzed using spatial multiomics to evaluate how cells interact with materials or respond to stimuli, such as mechanical loading, and create tools for the precise delivery of cells and molecules, mimicking the native tissue architecture. Its future directions can be tailoring regenerative approaches to individual patients based on their unique molecular and spatial tissue profiles, recreating functional and precise stem cell niches in vitro for disease modeling, drug screening and transplantation, and, with the help of artificial intelligence and computational modeling, even predict tissue regeneration outcomes and optimize bioengineered solutions, to name some expectations.

Although each of these spatial omics methods has its advantages and disadvantages, they can complement each other and provide a more comprehensive understanding of tissue biology. By combining these techniques, researchers can gain valuable insights into the spatial organization and function of tissues, which can help develop more effective tissue engineering strategies, even if they do not directly address biomechanical or tissue engineering aspects.
